# Outcomes after superficial temporal artery–middle cerebral artery anastomosis combined with multiple burr hole surgery and dural inversion synangiosis for moyamoya disease in adults

**DOI:** 10.3389/fsurg.2022.1047727

**Published:** 2022-11-04

**Authors:** Dongxiao Xu, Bingjie Zheng, Qiaowei Wu, Jinbiao Yao, Tatiana Ilyasova, Aferin Beilerli, Huaizhang Shi

**Affiliations:** ^1^ Department of Neurosurgery, The First Affiliated Hospital of Harbin Medical University, Harbin, China; ^2^ Department of Internal Diseases, Bashkir State Medical University, Ufa, Russia; ^3^ Department of Obstetrics and Gynecology, Tyumen State Medical University, Tyumen, Russia

**Keywords:** moyamoya disease, combined bypass, cerebral revascularization, superficial temporal artery-middle cerebral artery bypass, multiple burr hole, dural inversion

## Abstract

**Objective:**

Several forms of cerebral revascularization have been carried out to treat moyamoya disease, however, the existing methods are accompanied by a variety of complications. In this study, the authors aimed to evaluate the clinical and angiographic outcomes of a new surgical procedure: superficial temporal artery–middle cerebral artery (STA-MCA) anastomosis combined with multiple burr hole (MBH) surgery and dural inversion synangiosis for the treatment of moyamoya disease in adults.

**Methods:**

Patients treated for moyamoya disease from August 2019 to July 2021 were retrospectively reviewed. Clinical data, including perioperative complications and follow-up outcomes, were noted. Preoperative and postoperative angiograms were compared, and the diameters of the frontal branch of the superficial temporal artery (F-STA), the deep temporal artery (DTA), the distal superficial temporal artery (STA) before the bifurcation and the middle meningeal artery (MMA) were measured on preoperative and postoperative angiograms. Meanwhile, a Matsushima score was assigned from postoperative angiograms.

**Results:**

This study included 66 patients (67 hemispheres). During the follow-up period, a median of 18 (IQR, 13–21) months, no stroke or death occurred in any of the patients. The clinical outcomes were excellent in 27 patients (40.9%), good in 34 patients (51.6%), fair in 4 patients (6.0%), and poor in 1 patient (1.5%); the overall rate of favorable clinical outcomes (excellent and good) was 92.5%. The modified Rankin Scale (mRS) score was significantly improved at follow-up (*P* < 0.001). There were 41 hemispheres imaged by cerebral angiography after the operation, at a median postoperative interval of 9 (IQR, 8–12) months; among them, 34 (82.9%) hemispheres had Matsushima scores of grade A and grade B. The average postoperative diameters in the STA, DTA and MMA were increased significantly in 41 hemispheres at follow-up (*P* < 0.001). Sixteen (24.2%) patients suffered from perioperative complications, including focal hyperperfusion syndrome (HS) in 8 (12.2%) patients, cerebral infarction in 3 (4.5%) patients (including one case accompanied by wound infection), cerebral hemorrhage in 2 (3.0%) patients, seizures in 2 (3.0%) patients, and subdural effusion in 1 (1.5%) patient.

**Conclusions:**

The procedure of STA-MCA anastomosis combined with MBH surgery and dural inversion synangiosis may be a safe and effective treatment for adult patients with moyamoya disease.

## Background

Moyamoya disease (MMD) is a chronic occlusive cerebrovascular disease characterized by progressive stenosis or occlusion of the intracranial internal carotid arteries and/or their proximal branches, with an abnormal vascular network at the base of the brain and the basal ganglia (1, 2), leading to insufficient blood flow and chronic brain hypoperfusion (3).

The benefits of surgical revascularization, including direct bypass, indirect bypass and combined bypass, are currently acknowledged by neurosurgeons worldwide. It is worth noting that direct bypass procedures, such as superficial temporal artery–middle cerebral artery (STA-MCA) anastomosis, can immediately improve cerebral blood flow (CBF) and prevent stroke after surgery. Indirect bypass procedures, such as encephalo-duro-arterio-synangiosis (EDAS), encephalo-myosynangiosis (EMS), encephalo-duro-arterio-myo-synangiosis (EDAMS), multiple burr hole (MBH) surgery and dural inversion, can induce neovascularization between the brain surface and vascularized donor tissues, such as the dura mater and temporal muscle (4, 5). Combined bypass includes both direct and indirect bypass procedures, such as STA-MCA anastomosis combined with EDAS or STA-MCA anastomosis combined with EDAMS; combined bypass procedures are currently common in clinical use. At present, a growing number of studies suggest that combined bypass surgery is superior to direct or indirect bypass alone for the general MMD population because the surgical effect is secured by two means: an instant increase in blood flow from direct anastomosis and subsequent spontaneous ingrowth of collaterals from indirect bypass (6–9). The combined bypass procedures mentioned above are always associated with various postoperative complications, such as seizures, cerebral infarction, cerebral hemorrhage, etc. (10–12); therefore, we modified the combined bypass procedure to STA-MCA anastomosis combined with MBH surgery and dural inversion synangiosis. In this study, we will assess the efficacy of this surgical procedure by evaluating the improvement in clinical outcomes, modified Rankin scale (mRS) scores and angiographic changes in the treatment of MMD.

## Methods

This study was approved by the ethics committee of First Affiliated Hospital of Harbin Medical University, and informed consent was obtained from all participants or their legal representatives.

### Patient selection

This report was a retrospective study, and all patients who received STA-MCA anastomosis combined with MBH and dural inversion synangiosis at the First Affiliated Hospital of Harbin Medical University between July 2019 and August 2021 were eligible for the study. The inclusion criteria were as follows: (1) patients were over 18 years of age; (2) the diagnosis of MMD was confirmed by digital subtraction angiography; and (3) the hemisphere to be operated on first was the one causing symptoms of MMD. The exclusion criteria were as follows: (1) patients were under 18 years of age; (2) patients had secondary moyamoya syndrome caused by Down syndrome, neurofibromatosis, autoimmune disease, sickle cell disease, or a history of head irradiation.

Symptomatic hyperperfusion syndrome (HS) was defined as a significant increase in cerebral blood flow (CBF) at the anastomotic site, leading to significant neurological signs. HS can be divided into two categories: focal HS with transient neurological deficits and HS-induced intracranial hemorrhage. CT perfusion examination was performed in patients with complications to distinguish focal hyperperfusion syndrome from local hypoperfusion. CT or magnetic resonance imaging (MRI) was used to identify intracranial hemorrhage and cerebral infarction.

### Anesthetic considerations and surgical technique

Intraoperatively, the anesthesia team maintained strict control of blood pressure to avoid hypotension. Generally, the systolic blood pressure was kept at approximately 120 mmHg throughout the operation. Meanwhile, the anesthesiologist was also instructed to avoid hyperventilation, given the risk of vasoconstriction induced by hypocapnia, and to maintain the end-tidal pressure of CO_2_ between 35 and 45 mmHg.

**Figure 1:**
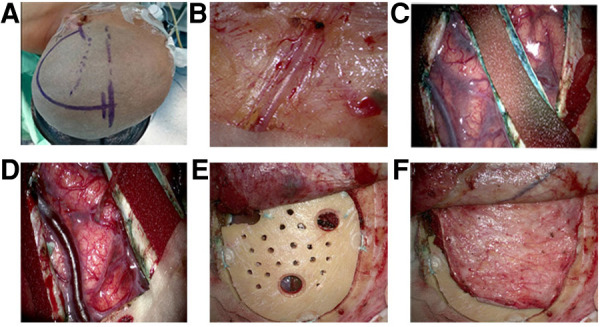
Surgical procedures. (**A**) Position and incisiona fronto-temporal-parietal approach was adopted, and the frontal branch of the (superficial temporal artery) STA was preserved. (**B**) The parietal branches of the STA was clearly visible on the medial surface of the flap and was carefully dissected from the flap as a donor vessel. (**C**) After the bone window was performed, the (middle meningeal artery) MMA trunk was preserved intact and incised on both sides. Then the remaining part of the dura mater was then opened radially, the margins inverted and tucked below the margins of the craniotomy. (**D**) The parietal branch of the separated STA was anastomosed to a recipient vessel (M4 segment of middle cerebral artery). (**E**) The free bone flap with multiple holes was returned to the anatomical position. (**F**) The temporal muscle flap was used to cover bone flap.

All patients were operated on by the same surgical procedure: STA-MCA anastomosis with MBH and dural inversion. Among the 66 patients, only 1 patient had surgery on both hemispheres. Since some patients had only unilateral symptoms and CT perfusion showed that there was no severe cerebral ischemia on the asymptomatic side, or the other side had already been subjected to another surgical procedure, the operations were performed on only one hemisphere. Even if a hemisphere was in Suzuki stage V, if the patient had significant symptoms or obvious hypoperfusion on CT perfusion, the operation was still performed. After the STA was mapped by intraoperative vascular Doppler ultrasound, a frontotemporal-parietal skin incision was performed, and the parietal branch of the STA was carefully dissected out of the scalps as a donor vessel. The frontal branch of the STA was preserved, as it can provide collateral extracranial-to-intracranial collateral circulation. Then, a temporal muscular flap was cut, and a 7- to 8-cm craniotomy was performed, on which 3 burr holes (each having a diameter of approximately 9 mm) were distributed across the frontal, temporal and parietal regions. Then, 5 to 7 new burr holes, each having a diameter of approximately 2 mm, were drilled between the 3 abovementioned burr holes. This procedure ensured not only the stability of the free bone flap but also the chance of neovascularization between the brain surface and external vascular areas. The underlying dura was opened in a radial fashion, and the margins were inverted and tucked under the craniotomy bone margin. At the same time, it was important to preserve as many of the MMA branches as possible because the importance of MMA to neovascularization has been accepted. After the parietal branch of the separated STA was anastomosed to a recipient M4 vessel, intraoperative indocyanine green (ICG) angiography proved that the anastomosis was patent. Then, the free bone flap with multiple holes was returned to the anatomical position, and the temporal muscle flap was used to cover it (Figure 1).

### Follow-up and data collection

The patients were followed up by outpatient visits or telephone 6 months of postoperatively. The clinical symptoms of patients were noted and classified according to the following categories at the last follow-up (13): (1) excellent, where the preoperative symptoms [such as transient ischemic attacks (TIAs) or seizures] completely disappeared and the neurological deficits were resolved; (2) good, where the symptoms completely disappeared but mild neurological deficits remained; (3) fair, where the symptoms persisted, albeit less frequently; and (4) poor, where the symptoms remained unchanged or worsened.

The functional outcome was evaluated using the mRS at discharge and at follow-up. A good functional outcome was recorded with an mRS score of 0–2.

### Angiographic evaluation

**Figure 2:**
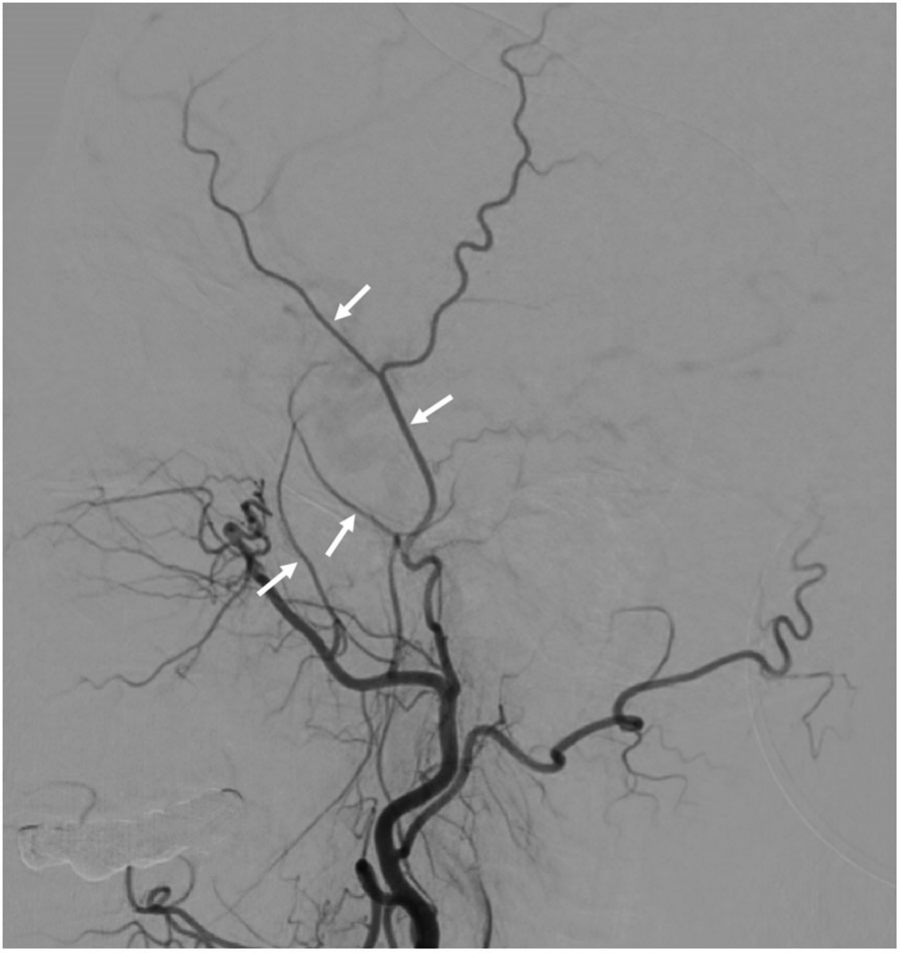
Example measurement sites for the superficial temporal artery (STA), the frontal branch of the STA (F-STA), the deep temporal artery (DTA) and middle meningeal artery (MMA) to allow comparison of preoperative and postoperative angiograms.

All patients underwent cerebral angiography preoperatively. Postoperative angiographic follow-up was scheduled after 6 months. In preoperative and follow-up angiograms, we used digital calipers to make measurements of the following structures on the lateral angiographic projection: the width of the distal STA before the bifurcation, the width of the proximal frontal branch of the STA (F-STA) just distal to the bifurcation, the width of the deep temporal artery (DTA), and the width of the MMA just distal to the foramen spinosum (Figure 2). Caliper measurements on preoperative and postoperative angiograms were made by 2 independent observers. The average of the 2 measurements was used for subsequent calculations and comparison. Additionally, the Suzuki grade and degree of collateralization were noted preoperatively, and collateral formation and Matsushima grade (as described below) were recorded postoperatively.

The development of collateral circulation of the middle cerebral artery (MCA) territory through the bypass was graded according to the system described by Matsushima et al., where a good score (A) represented revascularization of 2/3 of the MCA distribution, a fair score (B) represented revascularization of between 1/3 and 2/3 of the MCA distribution, and a poor score (C) represented slight or no revascularization (13).

### Statistical analysis

All data were analyzed using SPSS 22 (Statistical Package for the Social Sciences Software, IBM) for Windows. The measurement data that conformed to the normal distribution are presented as $\bar{x}\pm s$, and the categorical variables are presented as numbers of cases. The data with asymmetric distribution are expressed by the median (m) and the upper and lower quartiles (P25, P75). The Wilcoxon rank sum test was used for comparisons between groups. The intraclass correlation coefficient (ICC) was calculated to assess the intra-rater and interrater reliability. ICC values were graded using the following criteria (16): <0.5 suggests poor agreement; 0.5–0.75, moderate agreement; 0.75–0.90, good agreement and ≥0.90, excellent agreement. *P* < 0.05 was considered statistically significant.

## Results

### Clinical presentation

Sixty-six patients (67 hemispheres) underwent STA-MCA anastomosis combined with MBH surgery and dural inversion synangiosis, including 37 (56.0%) males and 29 (44.0%) females, with a mean age of 51.8 ± 8.3 years old (range, 32–69 years old). There were 54 (81.8%) patients who presented with ischemic symptoms, 8 (12.2%) patients with cerebral hemorrhage and 4 (6.0%) hemispheres with headache. Among these patients, 61 (92.4%) patients had an mRS score of 0–2, and 5 (7.6%) patients had a score of 3–4. In the 67 hemispheres treated with surgery, preoperative digital subtraction angiography (DSA) was used as a basis to apply the Suzuki staging system and divide the hemispheres into stages 1–6. Specific data are presented in Table 1.

**Table 1:** Clinical features.

Clinical Features	Number (%)
No. of patients	66
Age, years ($\bar{x}\pm s$)	51.8 ± 8.3
**Sex**	
Male	37 (56.0.)
Female	29 (44.0)
**Initial Symptoms**	
Hemorrhage	8 (12.2)
Cerebral infarction	22 (33.3)
TIA	22 (33.3)
Frequent TIA	10 (15.2)
Headache	4 (6.0)
**Comorbidity**	
Diabetes	15 (22.7)
Hypertension	38 (57.6)
Smoking	28 (42.4)
Drinking	23 (34.8)
**Baseline mRS score**	
0	1 (1.5)
1	39 (59.1)
2	21 (31.9)
3	3 (4.5)
4	2 (3.0)
Follow-up, months (IQR)	18 (8–12)
No. of treated hemispheres	67
**Suzuki stage**	
2	4 (6.0)
3	29 (43.3)
4	25 (37.3)
5	9 (13.4)

### Perioperative complications

Perioperative complications occurred in 16 (24.2%) patients, including focal hyperperfusion syndrome (HS) in 8 (12.2%) patients, cerebral infarction in 3 (4.5%) patients (one of whom also had a wound infection), cerebral hemorrhage in 2 (3.0%) patients, seizures in 2 (3.0%) patients, and subdural effusion in 1 (1.5%) patient. Among the 8 patients with hemorrhagic moyamoya disease, 1 (12.5%) had a recurrent cerebral hemorrhage. Specific data are presented in Table 2.

**Table 2:** Perioperative complications.

Complication	Number of patients (%)
Focal HS	8 (12.2)
Infarction	2 (3.0)
Hemorrhage	2 (3.0)
Seizures	2 (3.0)
Subdural effusion	1 (1.5)
Wound infection and infarction*	1 (1.5)

HS, hyperperfusion syndrome.

* Combined with cerebral infarction.

Among the 16 patients, 15 (22.7%) patients had a decline in functional status, including 11 (16.7%) patients whose mRS scores increased by 1 point, 2 (3.0%) patients who increased 2 points, and 2 (3.0%) patients who increased 3 points for a final score of 4. The other 51 (77.3%) patients showed a reduction or no change in their mRS scores. There was no significant difference in mRS scores before surgery and at discharge (*P* = 0.133).

### Clinical outcomes at follow-up

All patients were followed up with a median duration of 18 (IQR, 13–21) months, and no new-onset stroke occurred. For patients whose mRS score was increased by 2 or 3 at discharge, the mRS score returned to the preoperative level by the follow-up visit. Five (7.6%) patients had a decline of 2 points in their mRS scores from preoperative evaluation to follow-up; 40 (60.6%) patients lost 1 point; 19 (28.8%) patients had no change in their mRS scores; and only 2 (3.0%) patients, both of whom had postoperative hemorrhage, gained 1 point. Compared with preoperative scores, mRS scores were improved significantly at follow-up (*P* < 0.001).

Among the 66 patients, the postoperative clinical outcomes at follow-up were excellent for 27 (40.9%) hemispheres, good for 34 (51.6%) patients, fair for 4 (6.0%) patients, and poor for 1 (1.5%) patient. The overall satisfactory rate (excellent + good) was 92.5%. Among the patients with unsatisfactory clinical outcomes, 4 patients suffered from perioperative complications.

### Angiographic results

There were 41 patients (41 hemispheres) who underwent cerebral angiograms postoperatively. The ICC analysis demonstrated almost perfect intra-rater reliability (0.996–0.998, *P* < 0.001) of the diameters of arteries measurements. Besides, excellent agreement was found between the two readers with an ICC of 0.996 (*P* < 0.001), 0.996 (*P* < 0.001), 0.997 (*P* < 0.001) and 0.980 (*P* < 0.001) for the STA, F-STA, MMA and DTA measurements, respectively. There was a remarkable improvement in the average STA, F-STA, MMA and DTA diameters. The distal STA increased by an average of 34.8% (*P* < 0.001). The F-STA increased by an average of 23.2% (*P* < 0.001). The MMA increased by an average of 51.0% (*P* < 0.001). The DTA increased by an average of 29.3% (*P* < 0.001). There were 2 patients who showed a decline in F-STA vessel size after surgery, but their other vessel size demonstrated a significant increase. The F-STA vessel size of one patient changed by −32.9%, but the patient had a dramatic increase of 37.8% in the size of the parietal branches of the superficial temporal artery (P-STA); in addition, the size of the MMA increased by 86.0%. The F-STA vessel size of the other patient changed by −7.5%, while the size of the P-STA increased by 56.4%. Specific data are presented in Table 3.

**Table 3:** Measurements of change in vessel size.

Vessel	Preoperative	Postoperative	Change, %	*P*
DTA	0.75 ± 0.16	0.97 ± 0.20	29.3	<0.001
F-STA	0.95 ± 0.24	1.17 ± 0.34	23.2	<0.001
STA	1.38 ± 0.24	1.86 ± 0.34	34.8	<0.001
MMA	1.02 ± 0.28	1.54 ± 0.32	51.0	<0.001

F-STA, frontal branch of the superficial temporal artery; DTA, deep temporal artery; MMA, middle meningeal artery; STA, superficial temporal artery.

**Figure 3:**
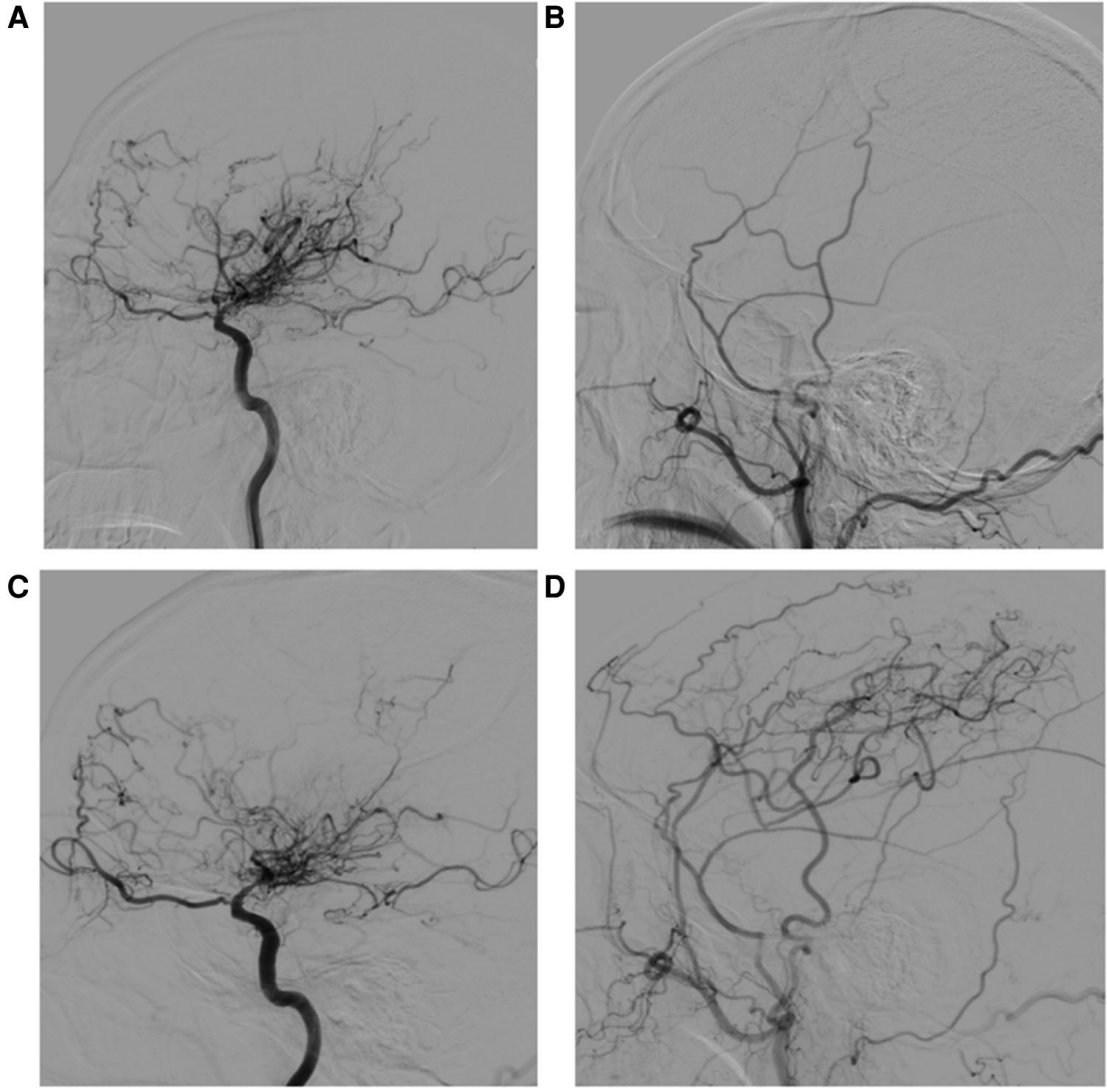
A 55-year-old man whose initial symptom was cerebral infarction. (**A**) Preoperative right internal carotid artery (ICA) angiogram (lateral view) reveals distal internal carotid artery occlusion with moyamoya collaterals. (**B**) Preoperative external carotid artery injection. (**C**) Postoperative right ICA angiogram (lateral view) 11 months after STA-MCA anastomosis with MBH and dural inversion showing decreased moyamoya vessels. (**D**) Early arterial-phase postoperative external carotid artery injection. Patency of the STA-MCA anastomosis was noted, and marked neovascularization associated with the frontal branch of the superficial temporal artery (F-STA), the deep temporal artery (DTA) and middle meningeal artery (MMA) was observed. The distal STA before the bifurcation increased in size by 43.2%, the F-STA increased by 10.2%, the DTA increased by 100%, and the MMA increased by 24.1%.

According to the postoperative DSA, the distribution of Matsushima scores for 41 patients was as follows: there were 22 patients with grade A, 12 with grade B and 7 with grade C, for an overall satisfactory rate of 82.9%. The 7 patients with a Matsushima score of C achieved satisfactory clinical outcomes nonetheless. All patients demonstrated collateral vessels through burr holes from the external carotid artery (ECA), with the DTA and F-STA being the predominant targets, but 3 patients had dissatisfactory results. Postoperative patency of the STA-MCA anastomosis was noted, except in 5 patients, whose clinical outcome was satisfactory. Interestingly, although the anastomosis patency of the two hemispheres was occluded, better Matsushima scores (grade A and grade B) were still achieved. An example case is shown in Figure 3.

## Discussion

Despite some controversy, revascularization is one of the main MMD treatments used to prevent recurrent stroke and to improve the prognosis (9, 10, 14). There are 3 types of ECA branches that can be used in these procedures: (1) the STA or occipital artery (OP), (2) the deep temporal artery (DTA) supplying temporal muscle and periosteum, and (3) the MMA supplying the cerebral dura mater. STA can be used in both direct and indirect revascularization procedures, such as STA-MCA anastomosis, encephalo-arterio-synangiosis (EAS), EDAS, and EDAMS. The DTA can be mainly used in EMS, EDAMS and MBH. MMA can be used in dural inversion, but there are few reports on the use of this procedure alone. Noshiro S et al. demonstrated that combined bypass takes advantage of long-term outcomes regarding clinical, angiographic, and hemodynamic statuses (15). In this study, we designed a new procedure of combined bypass to facilitate STA, DTA and MMA participation in revascularization.

### Advantage of STA-MCA anastomosis combined with MBH and dural inversion synangiosis

Our procedure was different from previously reported combined surgical procedures. Compared with other procedures, such as STA-MCA with EDAMS, our new procedure not only effectively used the branches of the external carotid artery but also achieved anatomical reattachment. The temporal muscle was cut above the bone flap to avoid creating a linear indentation that could cause aesthetic complaints. Additionally, this placement can reduce the neurologic deterioration resulting from the swelling of the temporal muscle, which contributes to the compression of the edge of the free bone flap (16). In our study, there were 2 patients who suffered from postoperative seizures, and the incidence was approximately 3.0%, which was lower than in other studies (10). The reason may be associated with a lack of compression of the temporal muscle. In these cases, we used only the parietal branch of the STA as the donor vessel for anastomosis with MCA and protected the frontal branch well, so that not only reduces the risk of wound infection (rate was 1.5%) but also allows the F-STA to better participate in intracranial blood supply. On the postoperative angiograms, we noted new revascularization from the branches of the ECA to each hemisphere. In this study, the diameter of the F-STA increased by an average of 23.2% (*P* < 0.001), the diameter of the MMA increased by an average of 51.0% (*P* < 0.001), the diameter of the DTA increased by an average of 29.3% (*P* < 0.001), and the diameter of the distal STA increased by an average of 34.8% (*P* < 0.001). According to the data above, MMA seems to be the most effective choice for neovascularization induced by indirect revascularization, which is consistent with other studies (17–19). Although the incidence of perioperative complications was 24.2%, most of them were reversible, and no patient experienced stoke recurrence after the first postoperative month. This suggests that patients develop enough new circulation to relieve ischemia and to significantly reduce the hemodynamic stress on the abnormal moyamoya vessels.

### Causes of complications

In our study, there were 16 patients who suffered from complications, and the incidence was 24.2%, which was significantly higher than the rates reported in other studies (20). The main reason is that we included patients with reversible functional impairment. These perioperative complications are closely related to the hemodynamic instability that characterizes MMD and the sudden CBF increase or other hemodynamic changes that can result from surgery (21). In our study, all complications occurred within two weeks after surgery. Three patients were diagnosed with cerebral infarction by MRI, and 2 patients were diagnosed with cerebral hemorrhage by CT; these complications resulted in neurological deficits. Many studies have indicated that postoperative ischemia may be associated with hypercapnia, hypotension and inadequate hematocrit (22, 23); however, hemorrhage is caused by hyperperfusion. Therefore, during the perioperative period, the systolic blood pressure was kept at approximately 120 mmHg, and hematocrit was adjusted to normal standards. The anesthesiologist was also instructed to avoid hyperventilation and maintain the end-tidal pressure of CO_2_ between 35 and 45 mmHg. Aside from the 5 above mentioned patients, in order to identify both focal hyperperfusion syndrome and local hypoperfusion, other patients with complications were examined for CT perfusion, from which we could detect a significant increase in intracranial blood flow, and the neurological deficits gradually recovered after medication was given to improve cerebral edema. According to the above, it is sensible to attribute other complications (excluding wound infection) to focal HS, which is consistent with the idea that HS is the most common complication in direct bypass and combined bypass (24). Postoperative seizures after bypass surgery are a common clinical presentation, but the phenomenon has rarely been discussed. Jin SC et al. considered that the occurrence of postoperative seizures was mainly related to synangiosis, early seizures were associated with increased bypass blood flow and HS, and advanced seizures were associated with increased cerebral cortex excitability caused by increased blood flow (25). In our series, there were 2 patients who suffered from seizures, which occurred on the 3rd and 7th days after surgery and were therefore classified as early seizures. Jin SC et al. (25) also considered that selecting frontal branches of the MCA as a recipient for STA-MCA anastomosis was taken as a risk factor for postoperative seizures. Therefore, we rarely used the frontal branches of the MCA as the recipient artery, which might be why the incidence of postoperative seizures was lower than in other studies.

### Factors contributing to lack of neovascularization

In this series, not all the patients had effective neovascularization. There were 2 patients with both ineffective neovascularization at burr holes and anastomotic non-patency, 3 patients with anastomotic non-patency alone, and 7 patients with a Matsushima score of C. Although these patients had ineffective neovascularization, their clinical outcome was satisfactory. Many factors may illuminate the lack of neovascularization. First, cerebral ischemia is considered the main driving force that induces neovascularization (12); meanwhile, Nakamura et al. (26) and Sainte-Rose et al. (27) confirmed that cerebral ischemia was essential for neovascularization induced by revascularization procedures in MMD patients. In our series, although 2 patients had both ineffective neovascularization at burr holes and anastomotic non-patency, they still had a satisfactory prognosis because they had good circulatory compensation from the anterior or posterior circulation. Therefore, it was supposed that these ineffective areas did not have enough demand for blood supply to drive neovascularization. In addition, there is a “competitive relationship” between direct and indirect bypasses, as well as between vessels in an indirect bypass. Although they can work together to supply blood to the cerebrum, which is the advantage of combined bypass procedures, they also compete with each other. Because the brain needs a limited amount of blood flow, when the indirect bypass has high flow, the direct bypass has low flow, and vice versa. In our study, 2 patients showed a decline in the size of F-STA vessels after surgery, but the sizes of other vessels (including MMA and P-STA) demonstrated a significant increase. Therefore, it was supposed that there was a “competitive relationship” between the direct and indirect bypasses. For some patients, direct bypass plays a major role in the early postoperative period, and indirect bypass plays a major role in the later period after the formation of indirect vascular anastomosis. When the intracranial vascular pressure was equal to or greater than that of the P-STA, the anastomosis was not patent. Thus, it appears that the relationship between direct and indirect bypasses includes competition.

Considering the above factors that inhibit neovascularization, we consider it best to place the burr holes and dural inversion in the most ischemic area; this area can be identified by preoperative DSA and CT perfusion. Because the recipient vessels we used were always relatively large vessels in diameter, the area where they were located may not have been the most ischemic in all cases. Therefore, the direct and indirect bypasses could have played better complementary roles. In addition, we tried our best to protect the integrity of the temporal muscle vascular network and the branches of the MMA because they may be taken as the donor arteries for neovascularization, playing a complementary role for direct bypass.

### Limitations

Our study was limited by its retrospective nature, and the sample size was small. In addition, only a small number of patients received CTP examination during follow-up, lacking the comparison of preprocedural and postprocedural CTP results. Moreover, this study did not compare the procedure with other treatment modalities, and further comparative studies would be of great interest.

## Conclusions

The procedure of STA-MCA anastomosis combined with MBH surgery and dural inversion synangiosis may be a safe and effective treatment and can achieve favorable clinical outcomes for adult patients with MMD. This modified surgical procedure can make full use of the blood supply from the branches of the ECA, including the MMA; the frontal and parietal branches of the STA; and the DTA. Additionally, direct and indirect bypasses can complement each other.

## Data Availability

The original contributions presented in the study are included in the article/Supplementary Material, further inquiries can be directed to the corresponding author/s.
